# Lessons learned from reviewing a hospital’s disaster response to the hydrofluoric acid leak in Gumi city in 2012

**DOI:** 10.1186/s12873-021-00427-1

**Published:** 2021-03-22

**Authors:** Heejun Shin, Se Kwang Oh, Han You Lee, Heajin Chung, Seong Yong Yoon, Sung Yong Choi, Jae Hyuk Kim

**Affiliations:** 1 Environmental Health Center, Soonchunhyang University Gumi Hospital, Gumi, Republic of Korea; 2 Department of Emergency Medicine, Soonchunhyang University Hospital, Bucheon, 170, Jomaru-ro, Bucheon-si, Gyeonggi-do 14584 Republic of Korea; 3 Department of Emergency Medicine, Chungnam National University Hospital, Daejeon, Republic of Korea; 4 Department of Emergency Medicine, Soonchunhyang University Hospital, Cheonan, Republic of Korea; 5 Department of Emergency Medicine, Soonchunhyang University Hospital, Seoul, Republic of Korea; 6 Environmental Health Center, Department of Occupational and Environmental Medicine, Soonchunhyang University Gumi Hospital, Gumi, Republic of Korea; 7 Department of Emergency Medicine, Mokpo Hankook Hospital, Mokpo, Republic of Korea

**Keywords:** Disasters, Surge capacity, Hydrogen fluoride, Review

## Abstract

**Background:**

This study analyzed the characteristics of hydrogen fluoride-exposed patients (HFEPs) treated in the emergency department (ED) of a local university hospital, and reviewed the hospital’s disaster response according to space, staff, supplies, and systems (4Ss).

**Methods:**

This retrospective observational chart review and descriptive study included 199 HFEPs among 2588 total ED patients who visited a local university emergency medical center for treatment between September 27, 2012 and October 20, 2012, following a hydrofluoric acid leak at the Hube Globe factory in Gumi City, Republic of Korea. Descriptive results concerning the 4Ss were obtained by interviewing ED specialist staff physicians on duty during the study period. In accordance with American Burn Association criteria, patients requiring burn center referral were assigned to the major burn group (MBG) as severe condition.

**Results:**

During the acute phase (within 8 h after leak initiation), there were 43 patients in the ED, which was staffed with 3 doctors and 3 nurses, without 4S resources. Of these 43 patients, there were 8 HFEPs (100%) in the MBG and 0 in the non-MBG (NMBG). During the subacute phase (24 h after the acute phase), there were 262 patients in the ED including 167 HFEPs, of whom 45 (26.95%) were in the MBG and 122 (73.05%) were in the NMBG. The ED was then staffed with 6 doctors (3 on day shift and 3 on night shift) and 10 nurses (3 on day shift, 4 on evening shift, and 3 on night shift), and no 4S resources were available. Throughout the study period, no 4Ss were available. First, there was no expansion of ED space or secured disaster reserve beds. Second, there was no increase in manpower with duty time adjustments or duty relocation for ED working personnel. Third, there was no logistics reinforcement (e.g., antidote or personal protective equipment). Fourth, there were no disaster-related measures for the administration department, decontamination zone setup, safety diagnostic testing, or designated disaster triage implementation.

**Conclusions:**

The hospital’s disaster response was insufficient for all aspects of the 4Ss. Detailed guidance concerning a hospital disaster management plan is required.

## Introduction

The cycle of disaster risk management has four stages: mitigation, preparedness, response, and recovery [1–4]. In terms of the disaster response, in conventional traumatic mass casualty incident (MCI) situations, patient severity classification and treatment prioritization are promoted for efficient resource distribution based on evaluation of the extent of physical injury, mainly by the medical resource provider [5]. However, in unique situations that correspond to chemical, biological, radiological, nuclear, and explosive (CBRNE) disasters, including previous considerations involved in conventional traumatic MCI, other concerns include zone setup, personal protective equipment (PPE), specialized triage, decontamination, and antidote management (if applicable) [6–8].

Gumi, Gyeongsangbuk-do, Republic of Korea is a state-governed industrial city with factories located in an industrial park [9, 10]. Chemical leaks have occasionally occurred in the community over the past several years [9–12]. These events include the Hube Globe factory leak of hydrogen fluoride (HF) in 2012, which garnered both domestic and international attention because of human and economic losses, as well as severe environmental damage involving crops [9, 12, 13]. The accident led to the creation of comprehensive chemical safety measures by the Ministry of the Environment and the establishment of the National Institute of Chemical Safety [14]. The HF leak impacted the local community, such that patients were sent to nearby local hospitals. However, those hospitals were unprepared for the disaster and had limited experience with zone setup, PPE, and decontamination agent supplies. They also lacked the capacity for expansion to manage the sudden surge in patients [9, 13, 15, 16].

In this study, we analyzed the basic demographic characteristics and clinical outcomes of HF-exposed patients (HFEPs) treated in the emergency department (ED) of a 400-bed university hospital, and conducted a literature review to examine the hospital’s response to a sudden surge of patients injured during an HF leak at a local factory.

## Methods

### Study population

Gumi, Gyeongsangbuk-do is Korea’s leading industrial city, with a population of 162,743 in September 2012 [17]. The population of interest in this study comprised 199 HFEPs who visited the ED at Soonchunhyang University Gumi Hospital due to the HF leak disaster at the Hube Globe factory in Gumi in 2012. Soonchunhyang University Gumi Hospital is a level II secondary hospital with approximately 39,000 annual patient visits to its ED. It is one of two main university hospitals in Gumi and has a total of 400 beds, including 20 ED beds. During the study period, 2588 patients visited the hospital’s ED.

### Inclusion criteria for HFEPs

All patients aged > 18 years who visited the ED of Soonchunhyang Gumi University Hospital due to HF exposure caused by the Hube Globe factory leak on September 27, 2012, were enrolled in the study.

### Inclusion criteria for all patients in the ED

All patients who visited the ED of Soonchunhyang University Gumi Hospital between September 27, 2012 and October 20, 2012 were included in the study.

### Study period

The study period was September 27, 2012 to October 20, 2012.

### Study design

This retrospective observational cross-sectional chart review and descriptive study was performed to examine the demographic and clinical characteristics of the patients and the disaster response of the hospital when confronted with a surge of patients following the HF leak. In addition, this study includes the results of an interview with an ED specialist staff physician who was on duty at Soonchunhyang University Gumi Hospital during the study period. This ED specialist staff physician was on duty in the late acute phase, early subacute phase, and part of the chronic phase during the study period. He was a representative interviewee on behalf of the other three ED specialist staff physicians who were on duty during the study period.

### Study population

#### Basic demographic characteristics and clinical outcomes of the 199 HFEPs according to the event timeline

The epidemiological and clinical outcome data of independent variables including age, sex, number of patients according to ED visit time, injury mechanism of patient, occupation, distance between patient location and incident location, injury severity, diagnosis, extent of damage, damaged site, and ED disposition were recorded and analyzed according to the event timeline. ED disposition was classified into four subgroups: discharge, discharge against medical advice (DAMA), death, and admission (ADM).

#### Specific progression comments of HFEPs corresponding to ED disposition subgroups

Specific progression comments of HFEPs corresponding to the four ED disposition subgroups are summarized in Table 2.

### Distribution and workload of ED manpower in the hospital for HFEPs, total patients, major burn group (MBG), and non-MBG according to the event timeline

#### Timeline of patient management (acute, subacute, and chronic phases)

Because the HF leak occurred at 4:00 pm on September 27, 2012, the first 8 h were defined as acute [9, 10, 12, 13], considering the time when patients who were directly exposed to HF in the factory or in areas adjacent to the factory visited the ED [9, 10, 12, 13]. The subacute phase was defined as the period when the HF gas spread widely in the air and affected the local community, during which time patients who felt symptoms during the 24 h from 0:00 am on September 28, 2012 to 0:00 am on September 29, 2012 most often visited the ED [9, 10, 12, 13]. The remaining period from 0:00 am on September 29, 2012 to 0:00 am on October 21, 2012 was defined as the chronic phase until no HFEPs visited the ED concerning the HF leak.

- Acute phase: 16:00 on September 27 to 00:00 on September 28, 2012 (first 8 h after HF leak)
- Subacute phase: 00:00 on September 28 to 00:00 on September 29, 2012 (24-h period after acute phase)
- Chronic phase: 00:00 on September 29 to 00:00 on October 21, 2012

#### Major burn criteria

Patients requiring burn center referral, in accordance with the American Burn Association criteria [18, 19], were assigned to the MBG. MBG inclusion criteria were as follows: partial thickness > 25% body surface area and age 10–50 years; partial thickness > 20% body surface area and age < 10 years or > 50 years; full thickness > 10% body surface area in any individual; burns involving hands, face, feet, or perineum; burns crossing major joints; circumferential burns of an extremity; burns complicated by inhalation injury; electrical burns; burns complicated by fracture or other trauma; or burns in high-risk patients who will require specialized social/emotional and/or long-term rehabilitative support, including cases involving suspected child abuse and substance abuse. Severely injured patients were assigned to the MBG, and all other patients were assigned to the non-MBG (NMBG). The MBG included one patient who was declared dead on arrival and one patient who died during treatment in the ED.

#### Distribution and workload of ED manpower for HFEPs, TPs, MBG, and NMBG according to the event timeline

The numbers of patients in the MBG and NMBG and the numbers of doctors and nurses on duty in the ED were investigated according to the timeline presented as Tables 1 and 3 in the Results section respectively. The patient per hour (PPH), defined as the number of patients per group/work hour × number of doctors or nurses, was regarded as the workload severity index. PPH load < 2.5 was defined as the optimal balance for the ED physician to prevent ED crowding, in accordance with the American Academy of Emergency Medicine policy statement [20]. PPH load < 1 for nurses managing patients requiring ventilator beds and PPH load < 2 for nurses managing patients requiring non-ventilator beds were defined as the optimal balance for ED staffing [18]. Work shifts for doctors were divided into day (8 am – 17 pm, 9 h) and night (17 pm – 8 am, 15 h), in accordance with the usual schedule. Work shifts for nurses were divided into day (7 am – 15 pm, 8 h), evening (15 pm – 23 pm, 8 h), and night (23 pm – 7 am, 8 h), in accordance with the usual schedule. The distribution and workload of ED manpower were confirmed by interviewing an ED specialist staff physician who was on duty at Soonchunhyang University Gumi Hospital during the study period.

### Treatment orders for HFEPs in the ED

Treatment orders issued in the ED were categorized according to their target (respiratory tract, skin burn, and systemic toxicity) based on the literature [19, 21, 22]. According to the target sites of the HFEPs, treatments based on calcium gluconate were performed in the ED [19, 21, 22]. Treatment orders for HFEPs were confirmed by medical chart reviews and by interviewing an ED specialist staff physician who was on duty during the late acute phase, early subacute phase, and part of the chronic phase during the study period.

### Checklist results of the hospital disaster response according to space, staff, supplies, and system

A checklist (Appendix) was developed comprising multiple questions to assess the hospital’s disaster response, mainly categorized into space, staff, supplies, and system (4Ss) after review of the literature [1–3, 5–11, 15, 16]. The answers were checked by medical chart review and by interviewing an ED specialist staff physician who was on duty during the study period.

### Statistical analyses

Data are expressed as the means ± standard deviations for continuous variables, and as frequencies (percentages) for categorical variables. *P*-values were calculated by one-way analysis of variance for continuous variables, and the chi-squared test with Yates’ continuity correction or Fisher’s exact test for categorical variables. Statistical analyses were performed with Rex (Version 4.0.2; RexSoft Inc., Seoul, Korea).

### Institutional review board approval

This study was supported by Soonchunhyang University and approved by the institutional review board of Soonchunhyang University Gumi Hospital (IRB_SCHUH 2019–18).

## Results

### Basic demographic characteristics and clinical outcomes of the 199 HFEPs according to the event timeline

The 132 (66.3%) male and 67 (33.7%) female HFEPs had a mean (standard deviation) age of 41.6 (14.4) years (Table 1). There were no differences in age and sex according to the event timeline (*p* = 0.3625 and *p* = 0.1983, respectively). In terms of mechanism, the proportions of inhalation injuries were higher than those of complex injuries (> 2) in all subgroups (*p* < 0.0001; Table 1). In terms of distance between patient location and incident location, 8 patients (100%) in the acute phase and 86 patients (51.50%) in the subacute phase were within 100 m from the incident location and more than 100 m away, respectively, but 15 patients (62.5%) in the chronic phase were less than 100 m away (*p* = 0.0062; Table 1). According to injury severity, the frequencies of non-major burns were highest in the subacute (122, 73.05%) and chronic (22, 91.67%) phases, but all 8 patients in the acute phase were in the MBG (*p* = 0.0001; Table 1). Chemical intoxication at diagnosis was observed in all phases, namely in 5 patients (62.5%) in the acute phase, 163 patients (97.6%) in the subacute phase, and 22 patients (91.67%) in the chronic phase (*p* = 0.0012; Table 1). Single-site damage occurred in 5 patients (62.5%) in the acute phase, 167 patients (100%) in the subacute phase, and 20 patients (83.33%) in the chronic phase (*p* < 0.0001; Table 1). Damage to the respiratory tract occurred in 5 patients (62.5%) in the acute phase, 163 patients (97.6%) in the subacute phase, and 22 patients (91.67%) in the chronic phase (*p* = 0.0001; Table 1). In terms of ED disposition, the DAMA group in the acute phase comprised 5 patients (62.5%), and the discharge group in the subacute and chronic phases comprised 167 patients (100%) and 23 patients (95.83%) (p < 0.0001; Table 1). Specific progression comments of HFEPs corresponding to the four subgroups of ED disposition are summarized in Table 2.

**Table 1:** Basic demographic characteristics and clinical outcomes of the 199 HFEPs according to the event timeline

Variable	Total (*n* = 199)	Acute phase (*n* = 8)	Subacute phase (*n* = 167)	Chronic phase (*n* = 24)	*P*-value
Age (years)	41.6 ± 14.4	36.63 ± 11.06	41.52 ± 14.22	44.08 ± 16.54	*0.3625
Sex					†0.1983
Male	132 (66.3%)	7 (87.50%)	112 (67.07%)	13 (54.17%)	
Female	67 (33.7%)	1 (12.50%)	55 (32.93%)	11 (45.83%)	
Injury mechanism					‡ < 0.0001
Inhalation	195 (97.99%)	5 (62.50%)	167 (100%)	23 (95.83%)	
Complex (more than two)	4 (2.01%)	3 (37.50%)	0 (0%)	1 (4.17%)	
Occupation					‡0.0021
Worker	48 (24.12%)	5 (62.50%)	39 (23.35%)	4 (16.67%)	
Resident	14 (7.04%)	0 (0%)	10 (5.99%)	4 (16.67%)	
Firefighter	17 (8.54%)	0 (0%)	17 (10.08%)	0 (0%)	
EMS technician	3 (1.51%)	0 (0%)	3 (1.80%)	0 (0%)	
Police	5 (2.51%)	0 (0%)	5 (2.99%)	0 (0%)	
Reporter	2 (1.01%)	2 (25.00%)	0 (0%)	0 (0%)	
Unknown	110 (55.28%)	1 (12.50%)	93 (55.69%)	16 (66.67%)	
Distance between patient location and incident location					‡0.0062
> 100 m	96 (48.24%)	0 (0%)	81 (48.50%)	15 (62.50%)	
≤ 100 m	103 (51.76%)	8 (100%)	86 (51.50%)	9 (37.50%)	
Injury severity					‡0.0001
MBG	55 (27.6%)	8 (100%)	45 (26.95%)	2 (8.33%)	
NMBG	144 (72.4%)	0 (0%)	122 (73.05%)	22 (91.67%)	
Diagnosis					‡0.0012
Chemical intoxication	190 (95.48%)	5 (62.50%)	163 (97.60%)	22 (91.67%)	
Complex (more than two)	9 (4.52%)	3 (37.50%)	4 (2.40%)	2 (8.33%)	
Extent of damage					‡ < 0.0001
Single site	192 (97.0%)	5 (62.50%)	167 (100%)	20 (83.33%)	
Multiple sites	7 (3.0%)	3 (37.50%)	0 (0%)	4 (16.67%)	
Damaged site					‡0.0001
Respiratory tract	188 (94.47%)	5 (62.50%)	163 (97.60%)	20 (83.33%)	
Complex (more than two)	11 (5.53%)	3 (37.50%)	4 (2.40%)	4 (16.67%)	
ED disposition					‡ < 0.0001
Discharge	191 (96.0%)	1 (12.50%)	167 (100%)	23 (95.83%)	
DAMA	5 (2.5%)	5 (62.50%)	0 (0%)	0 (0%)	
Death	2 (1.01%)	2 (25%)	0 (0%)	0 (0%)	
ADM	1 (0.5%)	0 (0%)	0 (0%)	1 (4.17%)	

Data are reported as the mean ± standard deviation for continuous variables and number (%) for categorical variables

*ADM* Admission, *Acute phase* First 8 h after onset of HF leak, *Subacute phase* 24 h after acute phase, *Chronic phase* 22-day period after acute and subacute phases, *DAMA* Discharge against medical advice, *ED* Emergency department, *EMS* Emergency medical service, *HFEPs* Hydrogen fluoride-exposed patients, *MBG* Major burn group, *NMBG* Non-major burn group

*One-way analysis of variance was performed

†Chi-squared test with Yates’ continuity correction was performed

‡Fisher’s exact test was performed

**Table 2:** Specific progression comments of HFEPs corresponding to ED disposition subgroups

ED disposition	Specific progression comment of HFEPs
Discharge	None specified
DAMA	Five patients in the DAMA subgroup in the acute phase comprised three factory workers and two news reporters. They received calcium gluconate nebulizer treatment for their dyspnea symptoms; ADM to the hospital was recommended, but they refused. Only one worker later visited the outpatient ophthalmology department.
Death	Two patients died in the acute phase, and had been diagnosed with complex injury (> 2) due to a combination of chemical intoxication and burns. One was dead upon arrival at the ED and the other was alive upon ED arrival but had severe hypocalcemia (blood calcium level, 3 mg/dL; normal adult range, 8.6–10.2 mg/dL) and recurrent refractory ventricular dysrhythmia. The patient died despite > 1 h of advanced cardiovascular life support, including calcium gluconate administration.
ADM	One patient was admitted to the hospital after visiting the ED with chief complaint of vomiting on October 6, 2012 after HF exposure, and was diagnosed with gastric ulcer. However, there were no definitive data in terms of criteria for ADM upon chart review.

*ADM* Admission, *HF* Hydrogen fluoride, *Acute phase* First 8 h after onset of HF leak, *Subacute phase* 24 h after acute phase, *Chronic phase* 22-day period after acute and subacute phases, *DAMA* Discharge against medical advice, *ED* Emergency department, *HFEPs* Hydrogen fluoride-exposed patients

### HFEPs and TPs in the ED by date

**Fig. 1:**
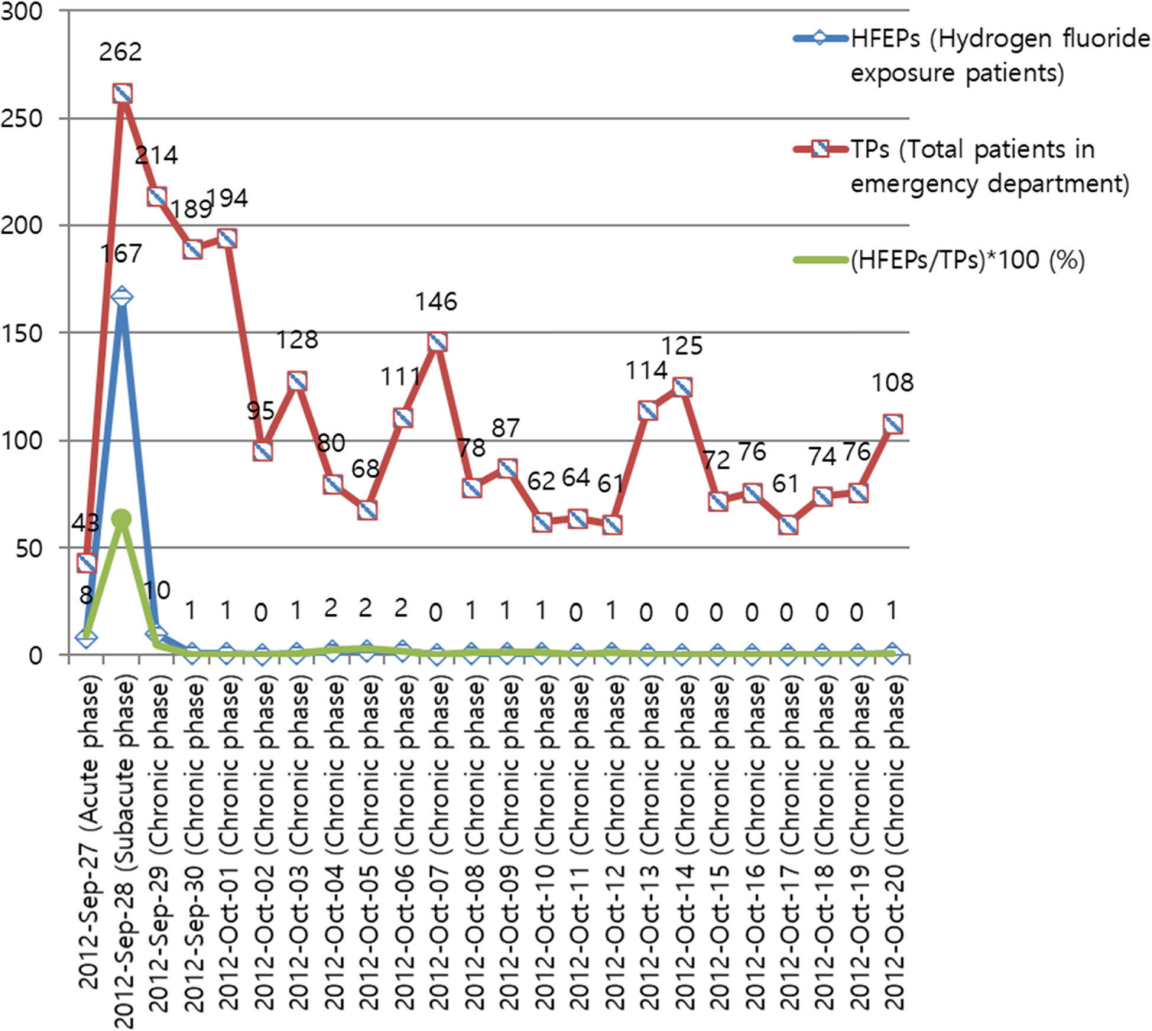
Frequencies of HFEPs and TPs in the ED by date. (HFEPs/TPs) × 100 (%), proportion of HFEPs among TPs in the ED; vertical axis represents the numbers of HFEPs and TPs, as well as the proportion of (HFEPs/TPs) × 100 (%)

Among the 2588 TPs, including the 199 HFEPs who visited the ED during the study period, 8 HFEPs (4.02%) and 43 TPs (1.66%) visited the ED during the acute phase. Thus, HFEPs comprised 9.64% of the acute-phase TPs (Fig. 1). During the subacute phase, 167 HFEPs (83.92%) and 262 TPs (10.12%) visited the ED, such that HFEPs comprised 63.74% of the TPs (Fig. 1). During the chronic phase, 24 HFEPs (12.06%) and 2283 TPs (88.21%) visited the ED, such that HFEPs comprised 20.35% of the TPs (Fig. 1).

### Distribution and workload of ED manpower in the hospital for HFEPs, TPs, MBG, and NMBG according to the event timeline

During the acute phase, eight patients in the MBG and no patient in the NMBG visited the ED, which was staffed at that time by three doctors and three nurses (Table 3). The PPH of TPs for doctors was highest at 1.86 for the night shift during the acute phase (Table 3). The PPH of TPs for nurses was highest at 2.33 for the night shift during the acute phase (Table 3). During the subacute phase, 45 patients in the MBG and 122 patients in the NMBG visited the ED (Table 3). The PPH of TPs for doctors was highest at 4.85 for the day shift during the subacute phase (Table 3). The PPH of TPs for nurses was highest at 4.88 for the day shift during the subacute phase (Table 3). During the chronic phase, 2 patients in the MBG and 22 patients in the NMBG visited the ED. No data were available regarding the number of doctors and nurses on duty in the ED during that time (Table 3). By interviewing an ED specialist staff physician who was on duty at Soonchunhyang University Gumi Hospital during the study period, we confirmed that there was no specialized ED triage for chemical disaster and no increase in manpower with duty time adjustments or duty relocation for HFEPs in any phase.

**Table 3:** Distribution and workload of ED manpower in the hospital for HFEPs, TPs, MBG, and NMBG according to the event timeline

**Acute phase (**16:00 pm September 27–00:00 am September 28, 8 h)											
Time	Work hour	Work shift	Doctor (n)	TPs (n)	HFEPs (n)	MBG (n)	NMBG (n)	PPH of TPs	PPH of HFEPs	PPH of MBG	PPH of NMBG
16:00 pm – 17:00 pm	1	Day	3	4	2	2	0	1.33	0.67	0.67	0
17:00 pm – 00:00 am	7	Night	3	39	6	6	0	1.86	0.29	0.29	0
Time	Work hour	Work shift	Nurse (n)	TPs (n)	HFEPs (n)	MBG (n)	NMBG (n)	PPH of TPs	PPH of HFEPs	PPH of MBG	PPH of NMBG
16:00 pm – 23:00 pm	7	Evening	4	76	19	7	19	1.29	0.68	0.25	0.68
23:00 pm – 00:00 am	1	Night	3	7	0	1	0	2.33	0	0.33	0
**Subacute phase (**00:00 am September 28–00:00 am September 29, 24 h)											
Time	Work hour	Work shift	Doctor (n)	TPs (n)	HFEPs (n)	MBG (n)	NMBG (n)	PPH of TPs	PPH of HFEPs	PPH of MBG	PPH of NMBG
00:00 am – 08:00 am	8	Night	3	58	47	45	2	2.42	1.96	1.88	0.08
08:00 am – 17:00 pm	9	Day	3	131	109	0	109	4.85	4.04	0	4.04
17:00 pm – 00:00 am	7	Night	3	73	11	0	11	3.48	0.52	0	0.52
Time	Work hour	Work shift	Nurse (n)	TPs (n)	HFEPs (n)	MBG (n)	NMBG (n)	PPH of TPs	PPH of HFEPs	PPH of MBG	PPH of NMBG
00:00 am – 07:00 am	7	Night	3	57	47	45	2	2.71	2.24	2.14	0.10
07:00 am – 15:00 pm	8	Day	3	117	101	0	101	4.88	4.21	0	4.21
15:00 pm – 23:00 pm	8	Evening	4	83	19	0	19	2.59	0.59	0	0.59
23:00 pm – 00:00 am	1	Night	3	5	0	0	0	1.67	0	0	0
**Chronic phase (**00:00 am September 29–00:00 am October 21, 22 days)											
Time	Work hour	Work shift	Doctor (n)	TPs (n)	HFEPs (n)	MBG (n)	NMBG (n)	PPH of TPs	PPH of HFEPs	PPH of MBG	PPH of NMBG
22 days	NA	NA	NA	2243	24	2	22	NA	NA	NA	NA

*ED* Emergency department, *HF* Hydrogen fluoride, *HFEPs* Hydrogen fluoride-exposed patients, *TPs* Total patients in emergency department, *MBG* Major burn group, *NMBG* Non-major burn group, *NA* Not available or not accountable, *PPH* Patient per hour (number of patients per group/work hour × number of doctors or nurses and considered the workload severity index, *Acute phase* First 8 h after onset of HF leak, *Subacute phase* 24 h after acute phase, *Chronic phase* 22-day period after acute and subacute phases, *Work shift* Work shifts for doctors were day (8 am – 17 pm, 9 h) and night (17 pm – 8 am, 15 h), and work shifts for nurses were day (7 am – 15 pm, 8 h), evening (15 pm – 23 pm, 8 h), and night (23 pm – 7 am, 8 h)

### ED treatment orders implemented for HFEPs

Treatment of the HFEPs, determined using ED treatment orders, was classified according to the target (respiratory tract, skin burns, and systemic intoxication; Table 4).

**Table 4:** Emergency department treatment orders implemented for HFEPs in the 2012 Gumi City HF leak disaster

Target site	Emergency department treatment order for HFEPs
Respiratory tract	Application of nebulizer with 2 mL mixed calcium gluconate solution comprising 1 ampoule calcium gluconate (2.084 g/20 mL) dissolved in 100 mL normal saline (0.9 g sodium chloride)
Skin burns	Application of gauze soaked with 1 ampoule calcium gluconate (2.084 g/20 mL) dissolved in 100 mL normal saline
Systemic intoxication	Intravenous administration of calcium gluconate (2.084 g/20 mL)

According to target sites of HFEPs, treatments based on calcium gluconate were performed in the emergency department [19, 21, 22]. Treatment orders for HFEPs were confirmed by medical chart reviews and by an interview with one representative emergency department specialist staff physician who was on duty during the late acute phase, early subacute phase, and part of the chronic phase during the study period

*HFEPs* Hydrogen fluoride-exposed patients, *HF* Hydrogen fluoride

### Checklist results for the hospital’s disaster response according to the 4Ss

In the staff category, there was no reinforcement of the hospital’s disaster response personnel or duty time adjustments or duty relocation for ED working personnel (Table 5). In the space category, there was no expansion of ED space to inside or outside of ED, or acquisition of disaster reserve beds (Table 5). In the supplies category, there was no reinforcement of medicines including antidote (e.g., calcium gluconate), provision of PPE, or implementation of other logistics required for the hospital’s disaster response (Table 5). In the system category, there were no changes in the hospital’s disaster command system or any process to invoke surge support, implement disaster-related measures in the administration department, or disaster triage activities (e.g., START or SALT or decontamination zone setup, or decontamination or disaster-related diagnostic testing measures, or unification and management of the entrances and exits of hospitals) (Table 5).

**Table 5:** Checklist results of hospital disaster response according to the 4Ss

Category	Question	Yes or No
Space	Was there any expansion of ED space to accommodate additional patients inside the ED?	^a^No
	Was there any expansion of ED space to accommodate additional patients outside the ED?	^a^No
	Were any disaster reserve beds secured in the hospital?	^a^No
Staff	Was there any reinforcement of hospital disaster response personnel (e.g., doctors or nurses), administration personnel, or security personnel?	^a^No
	Were there any duty time adjustments or duty relocation of ED working personnel?	^a^No
Supplies	Was there any reinforcement of medicines including antidote (e.g., calcium gluconate)?	^a^No
	Was there any personal protective equipment provided for hospital disaster response personnel to respond to the CBRNE disaster?	^a^No
	Were there any reinforcement of logistics other than those mentioned above for the hospital’s disaster response?	^a^No
System	Were any hospital disaster command systems in operation?	^a^No
	Was there any process to invoke surge support?	^a^No
	Did the administration department implement disaster-related measures to accept a larger number of patients than usual?	^a^No
	Was disaster triage (e.g., START or SALT in preparation for multiple casualty accidents or disasters) implemented in addition to the usual ED patient triage?	^a^No
	Was any decontamination zone established in the hospital?	^a^No
	Did the hospital perform decontamination of the patients?	^a^No
	Were any specialized diagnostic testing measures implemented to address the rapidly surging ED patient testing needs?	^a^No
	Was there any unification and management of the entrances and exits of hospitals that should be performed in disaster situations?	^a^No

*CBRNE* Chemical, biological, radiological, nuclear, and explosives, *ED* Emergency department

^a^We checked and confirmed these results by medical chart review and by interview with ED specialist staff physician who was on duty during the study period. We developed these checklist questions by reviewing literature concerning the hospital’s disaster response [1–3, 5–11, 15, 16]

## Discussion

There were no actions implemented in accordance with the 4Ss in this study. It is essential to plan how hospitals will utilize their own resources to adequately address the rapidly growing patient demand when a large number of injured individuals visit the ED due to a CBRNE disaster or MCI.

In reviewing the epidemiological and clinical characteristics of the 199 HFEPs, most visit the ED during the subacute phase and more than 80% visited the ED within 24 h after the onset of the incident. These results are similar to those of previous studies [23, 24]. Nearly half of the patients visited the ED due to inhalation complaints, had been 100 m away from the incident location, and were diagnosed with chemical intoxication. This diagnosis reflected the delayed evacuation of Gumi’s residents in the absence of governmental guidance, as described in two reports [12, 13]. All eight HFEPs assigned to the MBG based on injury severity presented to the ED during the acute phase, consistent with the results of a Centers for Disease Control and Prevention simulation study in which nearly all individuals with acute casualties arrived at the nearest ED within approximately 7 h [25]. Two patients in this group died. Concerning fatalities among highly concentrated HFEPs, severe instances of myocardial irritability, arrhythmia, and even death have been reported [26]. In the chronic phase, one HFEP was admitted to the hospital after visiting the ED with the chief complaint of vomiting on October 6, 2012, and was diagnosed with gastric ulcer. We postulate that HF exposure caused the gastric ulcer, in accordance with its published description: “strong acid that produces a high concentration of hydrogen ions, causing coagulative protein necrosis, and direct destruction of exposed tissues.” [26]. ED-generated orders for 199 HFEPs were produced and applied according to the target site, which comprised the respiratory tract, skin burn, and systematic intoxication, based on findings in previous studies [19, 21, 22]. The most prevalent injury site in this study was the respiratory tract. The evidence-based recommended treatment for respiratory HFEP was 2.5–3.0% calcium gluconate using a nebulizer with inhalation, as well as management of systemic toxicity [26]. However, to date, published studies have suggested various treatment protocols, and there is no widely accepted protocol for the treatment of HF burns [26–28].

In reviewing the hospital’s chemical disaster response according to 4Ss, there was no expansion of ED space to accommodate additional patients inside or outside the ED, and no acquisition of disaster reserve beds. Securing and expanding space in a chemical MCI situation is very important. In a bottleneck surge capacity prediction simulation conducted in MCI mode and involving burn patients, the lack of beds for critically injured patients was the first problem to emerge [29]. Application of the hospital acute care surge capacity disaster metric, defined as total ED beds/2.5, to the 20-bed ED of the hospital in our study, revealed that 8 beds per hour were needed [30]. Furthermore, assuming a 1:3 ratio of patients with severe and moderate needs, two patients in severe condition and six with moderate needs will require 1 h care in the ED, and 48 patients (12 with severe and 36 with moderate needs) will require 6 h care, corresponding to the acute treatment phase for trauma-related MCIs [30]. Thus, the maximum medical capacity of the ED must be determined carefully.

During progression from the acute phase to the subacute phase, the PPH of TPs and MBG for doctors and nurses increased above 4, which is beyond the optimal balance of ED staffing [18, 20]. For burn patients in MCI or CBRNE disasters, a nurse:patient ratio of 1:2–1:4 and a doctor:patient ratio of 1:50–3:50 are recommended [29]. To maintain the continuity of the standard of care in MCI or CBRNE disasters, hospitals must distinguish three levels of care situations: “conventional care” as usual care, “contingency care” as functionally equivalent care in a contingency situation, and “crisis care” as crisis standard of care for space, staff, and supplies such as the acquisition of disaster reserve beds by using non-patient care areas such as conference rooms for patient care [5]. ED staff require administrative support to manage patients efficiently by avoiding ED registration bottlenecks [1, 2, 5, 7]. It is important to separate patients with guardians, staff entry areas, triage areas, and parking lots, as well as to manage unified entrances and exits of hospitals in MCI or CBRNE disasters [1, 2, 5, 7]. Prepared offline registration methods such as numbered disaster patient tag necklaces or bracelets may be effective [1, 2, 5, 7]. To prepare for fatal situations, it is important that ED staff have the opportunity to make screening decisions and opportunities by using a simulation setting (on-line or off-line), tabletop exercises, courses with modules such as triage and discussion (e.g., MCI debriefing or additive actions needed in CBRNE disasters), or functional disaster exercises [5–8].

In the event of an MCI or CBRNE disaster, ED doctors should minimize diagnostic tests and focus on lifesaving procedures [5, 15, 16]. Furthermore, laboratory workers and radiologists must prioritize testing [5, 15, 16, 27]. Systems should be implemented to detect and reinforce the shortage or absence of medicines or supplies (e.g., PPE) for the hospital’s disaster response [5, 15, 16, 27]. Moreover, when faced with resource shortages caused by a surge in disaster demand, suppliers can use six key strategies: prepare, conserve (by resource restriction), substitute (by replacement with functionally equivalent items), adapt (by using items for unintended purposes), reuse (by cleaning and disinfection), and reallocate resources (as a last resort) [5]. Detailed preparation enables the identification and mitigation of resource shortages by planning, and maintenance of supplies by preparation [5]. Proactive measures for each level of care must be determined in advance and applied when appropriate [5, 31]. ED care providers should be aware of risks to the surrounding community through a hazard vulnerability assessment, understand the possible medical consequences, and provide education concerning CBRNE topics to the ED staff [5, 7, 16, 32]. For example, in an industrial city such as Gumi City, considering that various chemical substances are handled in factories, ED-based response training is necessary for scenarios in which chemical disasters occur because of extensive chemical leakage. Such training should include zone setup, decontamination, PPE level determination, and the use of antidotes, if applicable [5–8]. Disaster medical experts must be aware of the need for an integrated systematic approach to deal with CBRNE incidents, guided by seven key concepts related to effective disaster management: (1) basic and clinical sciences, (2) modeling and systems management, (3) planning, (4) response and incident management, (5) recovery and resilience, (6) lessons learned, and (7) continuous improvement [7].

To identify the best possible disaster triage model, Craig et al. [33] compared ED triage methods such as START, ESI, CBRN, and SALT using data extracted from the medical records of patients from the Graniteville chlorine disaster caused by train derailment in 2005. Determination of patient exposure to HF, and evaluating injury severity by early ED triage (mainly using initial vital signs, present illness, symptoms, and signs) was challenging with limited human resources and no expansion of ED space within a limited period for multiple HFEPs visiting the ED when HF inhalation constituted the main patient injury mechanism. One effort to address this challenge was conducted by Cully et al. [34], who retrospectively analyzed patient data from a chlorine leak disaster caused by train derailment in Graniteville, SC, USA in 2005. They determined that irritant gas syndrome agent exposure could be validated, and ED care should be given priority, if the patient met ≥2 clusters among 3 clusters of symptoms and signs which constituted respiratory, chest discomfort, and eye, nose and/or throat. Before the implementation of these disaster triages, the premise of judgment must be preceded by seven core ethical decision-making systems of fairness to all individuals and the process itself: duty to care, duty to steward resources, transparency of the process and criteria, consistency to all patients, proportionality in degree of resource restriction according to demand, accountability of triage officers and others to defend their decisions, and answering questions from others [5].

### Limitation

This study had the potential for selection bias, because patient information was collected retrospectively from a single institution. However, disasters are mainly studied in retrospective anecdotal case reviews in an environment that is not ethically feasible for a randomized controlled trial.

## Conclusion

The hospital’s disaster response was insufficient in all aspects of the 4Ss. Detailed guidance is necessary to establish a hospital disaster management plan.

## Data Availability

The data that support the findings of this study are available from the electronic medical record database of Soonchunhyang University Gumi Hospital (Southeastern Region of South Korea, Gyeongsangbuk-do, Gumi city), but these data were used under license for the current study, so they are not publicly available. However, data are available from the authors upon reasonable request and with permission from the given registry. Data access and analysis were approved by the institutional review board of Soonchunhyang University Gumi Hospital (IRB_SCHUH 2019–18).

## Appendix

**Table 6:** Checklist of the hospital’s disaster response according to space, staff, supplies, and system

Category	Question	Yes or No
Space	Was there any expansion of ED space to accommodate additional patients inside the ED?	Yes or No
	Was there any expansion of ED space to accommodate additional patients outside the ED?	Yes or No
	Were any disaster reserve beds secured in the hospital?	Yes or No
Staff	Was there any reinforcement of hospital disaster response personnel (e.g., doctors or nurses), administration personnel, or security personnel?	Yes or No
	Were there any duty time adjustments or duty relocation of ED working personnel?	Yes or No
Supplies	Was there any reinforcement of medicines including antidote (e.g., calcium gluconate)?	Yes or No
	Was there any personal protective equipment provided for hospital disaster response personnel to respond to the CBRNE disaster?	Yes or No
	Were there any reinforcement of logistics other than those mentioned above for the hospital’s disaster response?	Yes or No
System	Were any hospital disaster command systems in operation?	Yes or No
	Was there any process to invoke surge support?	Yes or No
	Did the administration department implement disaster-related measures to accept a larger number of patients than usual?	Yes or No
	Was disaster triage (e.g., START or SALT in preparation for multiple casualty accidents or disasters) implemented in addition to the usual ED patient triage?	Yes or No
	Was any decontamination zone established in the hospital?	Yes or No
	Did the hospital perform decontamination of the patients?	Yes or No
	Were any specialized diagnostic testing measures implemented to address the rapidly surging ED patient testing needs?	Yes or No
	Was there any unification and management of the entrances and exits of hospitals that should be performed in disaster situations?	Yes or No

We developed these checklist questions by reviewing literature concerning the hospital’s disaster response [1–3, 5–11, 15, 16]

*CBRNE* Chemical, biological, radiological, nuclear, and explosives, *ED* Emergency department

## Abbreviations

CBRNE: Chemical, biological, radiological, nuclear, and explosive
ED: Emergency department
HFEPs: Hydrogen fluoride-exposed patients
MCI: Multiple casualty incident
PPE: Personal protective equipment
TPs: Total patients in the ED
